# Vaccines targeting *Staphylococcus aureus* skin and bloodstream infections require different composition

**DOI:** 10.1371/journal.pone.0217439

**Published:** 2019-06-10

**Authors:** Brian M. Luna, Travis B. Nielsen, Brian Cheng, Paul Pantapalangkoor, Jun Yan, Susan Boyle-Vavra, Kevin W. Bruhn, Christopher Montgomery, Brad Spellberg, Robert Daum

**Affiliations:** 1 Department of Medicine, Keck School of Medicine at the University of Southern California (USC), Los Angeles, CA, United States of America; 2 Department of Molecular Microbiology & Immunology, Keck School of Medicine at the University of Southern California (USC), Los Angeles, CA, United States of America; 3 Loyola Stritch School of Medicine, Chicago, IL, United States of America; 4 Department of Medicine, University of Maryland, Baltimore, MD, United States of America; 5 Rosalind Franklin School of Medicine, Chicago, IL, United States of America; 6 National Institute of Health, Center for Scientific Review, Infectious Disease and Microbiology, Bethesda, MD, United States of America; 7 Research Institute at Nationwide Children’s Hospital, Columbus, OH, United States of America; 8 Department of Pediatrics, The Ohio State University College of Medicine, Columbus, OH, United States of America; Instituto Butantan, BRAZIL

## Abstract

*Staphylococcus aureus* infections represent a major public health threat, but previous attempts at developing a universal vaccine have been unsuccessful. We attempted to identify a vaccine that would be protective against both skin/soft tissue and bloodstream infections. We first tested a panel of staphylococcal antigens that are conserved across strains, combined with aluminum hydroxide as an adjuvant, for their ability to induce protective immunity in both skin and bacteremia infection models. Antigens were identified that reduced dermonecrosis during skin infection, and other non-overlapping antigens were identified that showed trends to protection in the bacteremia model. However, individual antigens were not identified that mediated substantial protection in both the skin and bacteremia infection models. We therefore tested a variety of combinations of proteins to seek a single combination that could mediate protection in both models. After iterative testing, a vaccine consisting of 3 antigens, ABC transporter protein (SACOL2451), ABC2 transporter protein (SACOL0695), and α-hemolysin (SACOL1173), was identified as the most effective combination. This combination vaccine provided protection in a skin infection model. However, these antigens were only partially protective in the bacteremia infection model. Even by testing multiple different adjuvants, optimized efficacy in the skin infection model did not translate into efficacy in the bacteremia model. Thus protective vaccines against skin/soft tissue infections may not enable effective protection against bloodstream infections.

## Introduction

*Staphylococcus aureus* is the most common cause of skin infections and the second most common cause of bacteremia [1–4]. The spread of community-associated methicillin-resistant *S*. *aureus* (MRSA) has made treatment of such infections challenging [5–8]. Given their high incidence and drug-resistance, a vaccine to prevent such *S*. *aureus* infections would have an enormous impact on US and global health.

Recent vaccine attempts have not yielded promising clinical results. Tefibazumab was developed as passive immunotherapy that targeted clumping factor A (ClfA) but showed poor efficacy in combination with antibiotics [9,10]. A phase II clinical trial involving V710 (Merck), a vaccine developed against iron-regulated surface determinant protein B (IsdB) was terminated early due to increased mortality among vaccinated patients [11]. StaphVax, a vaccine directed against the capsular polysaccharides CP5 and CP8, advanced to a phase III clinical trial but was also unsuccessful [12].

Many of these attempts have largely targeted single antigens rather than combinations of antigens (multivalent vaccine), which may be too simplistic with regards to the complex host-pathogen interactions that develop during infection. There is evidence that combinatorial strategies elicit greater protection than targeting of single antigens. A vaccine composed of IsdB, iron-regulated surface determinant protein A (IsdA), serine aspartate containing protein D (SdrD), serine aspartate containing protein E (SdrE) protected mice from lethal infection better than any of the single components [13]. However, underscoring the complexity of staphylococcal vaccination, clinical development of the quadrivalent vaccine (Pfizer) was terminated after a phase IIb trial demonstrated it did not reduce invasive infections following spinal surgery [14].

We hypothesized that active vaccines targeting *S*. *aureus* may require a combination of multiple antigens [15–17]. In order to support basic vaccinology research, Merck agreed to supply numerous recombinant proteins which they have internally demonstrated to have varying measures of protective efficacy against *S*. *aureus* infection in mice. We selected this panel of antigens, in combination with several novel candidates identified by the authors. We hypothesized that such a multivalent vaccine might protect the host against the two predominant infectious syndromes caused by *S*. *aureus*: bloodstream and skin. We also hypothesized that antigenic targets and host defense elements required for protection would differ during bloodstream and skin infections.

## Results

### Single antigen immunization

We tested each antigen alone to determine if the antigen was sufficient to provide protection in the bacteremia or skin infection models. Because antibody titers have not been found to be accurate surrogate markers of efficacy in prior studies in mice or humans,[15,16,18] we chose not to rely on antibody titers to select candidates to move forward. Rather, we used clinical endpoints to select candidate proteins. Specifically, we used survival time for the bacteremia model, which is a lethal model. The skin model does not produce a lethal infection and vaccine efficacy is defined as the development of smaller skin lesions (area).

**Fig 1:**
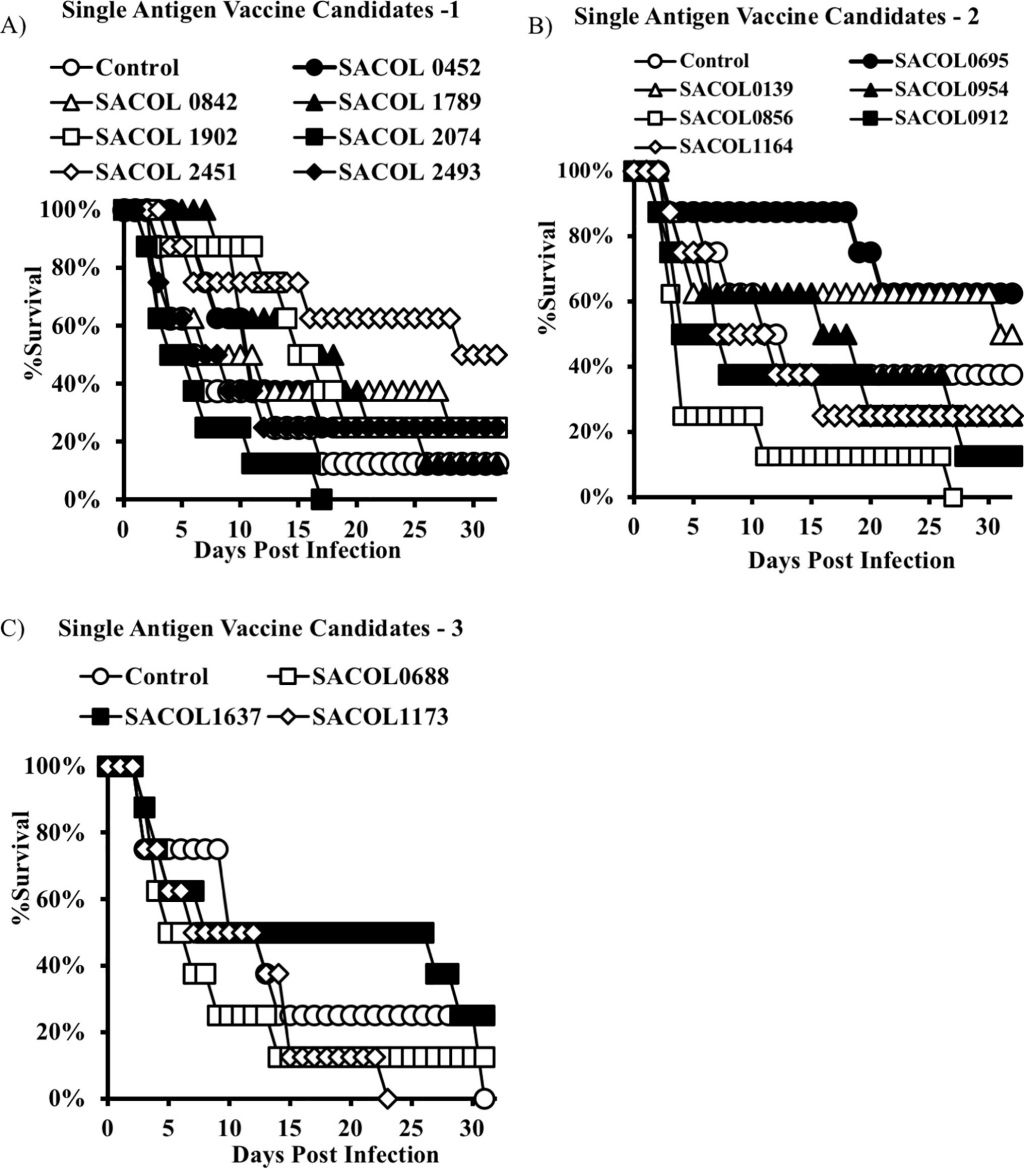
Single antigen immunization, bacteremia model. BALB/c mice (n = 8) were immunized with individual antigens (20 micrograms) and then challenged with a *S*. *aureus* IV infection. Mice were monitored daily and euthanized when moribund. Immunization with SACOL2451 and SACOL0695 resulted in the highest percentage of surviving mice.

**Fig 2:**
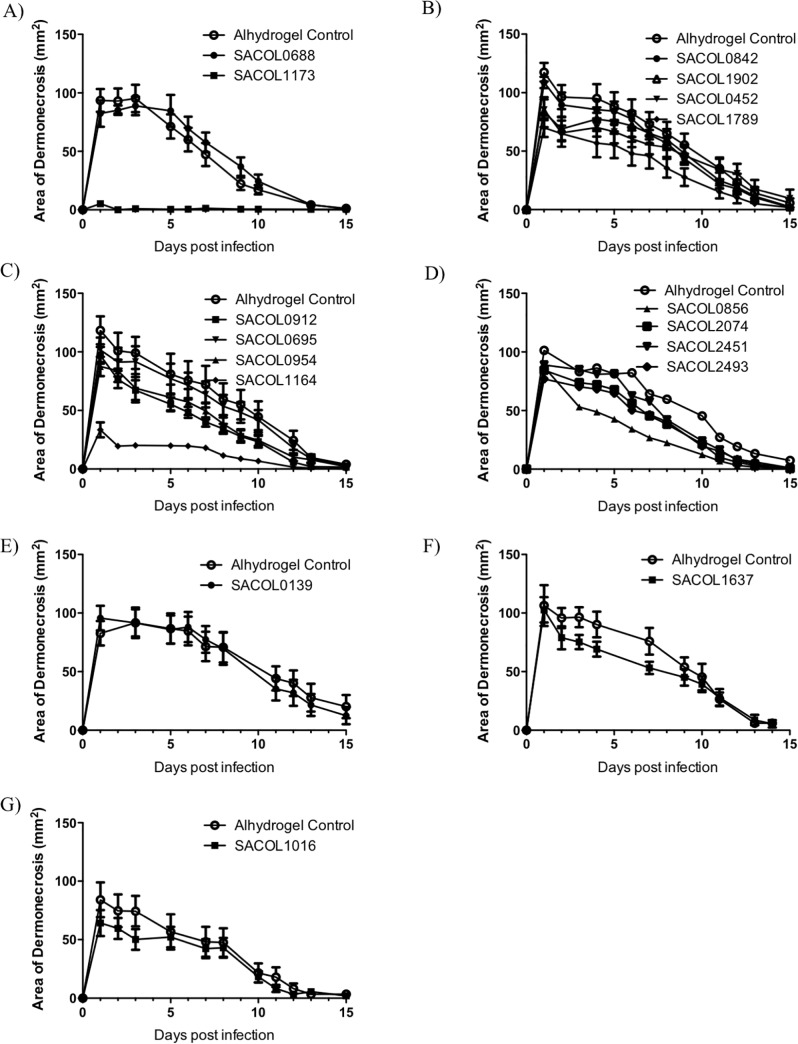
Single antigen immunization, skin model. BALB/c mice (n = 8) were immunized with individual antigens (20 micrograms) and then challenged with a *S*. *aureus* in a skin infection model. Mice were monitored daily and the wound size was recorded. The single antigens that provided the best protection in the skin model were SACOL1173 and SACOL1164.

We found minimal overlap in the antigens that were found to be protective in each respective model (Figs 1 and 2). For the bacteremia model, SACOL0695 and SACOL2451 provided the best trend to protection, although statistical significance was not achieved. However, for the skin model SACOL0856, SACOL1164, SACOL1173, and SACOL1789 provided significant protection (Fig 2).

**Fig 3:**
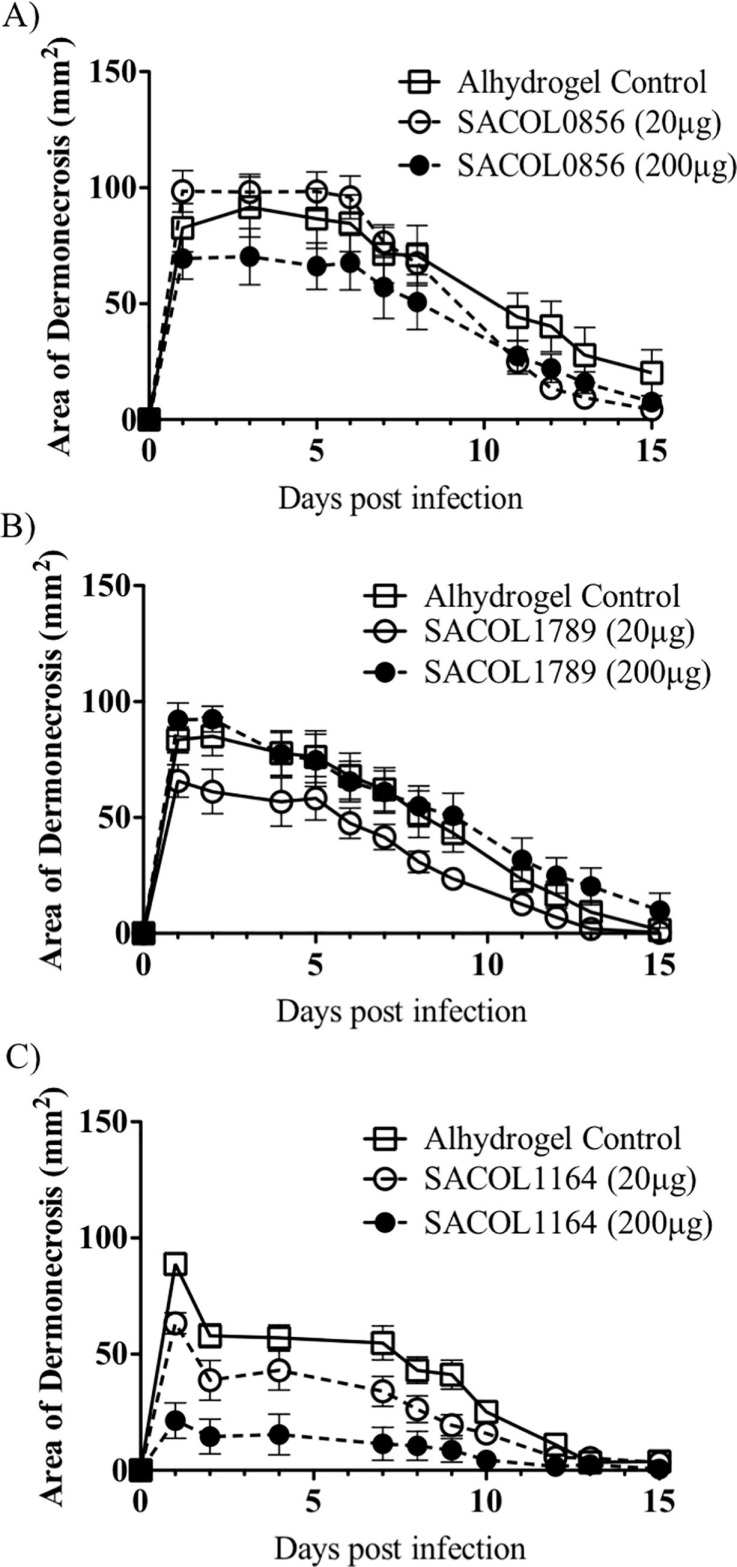
Dose optimization. **A-C)** Skin infection model. BALB/c mice (n = 8) were immunized with individual antigens that were found to be protective during the initial screen and then challenged with a *S*. *aureus* in a skin infection model. Mice were monitored daily and the wound size was recorded. Increasing the antigen dose 10-fold did not result in a statistically significant benefit.

**Fig 4:**
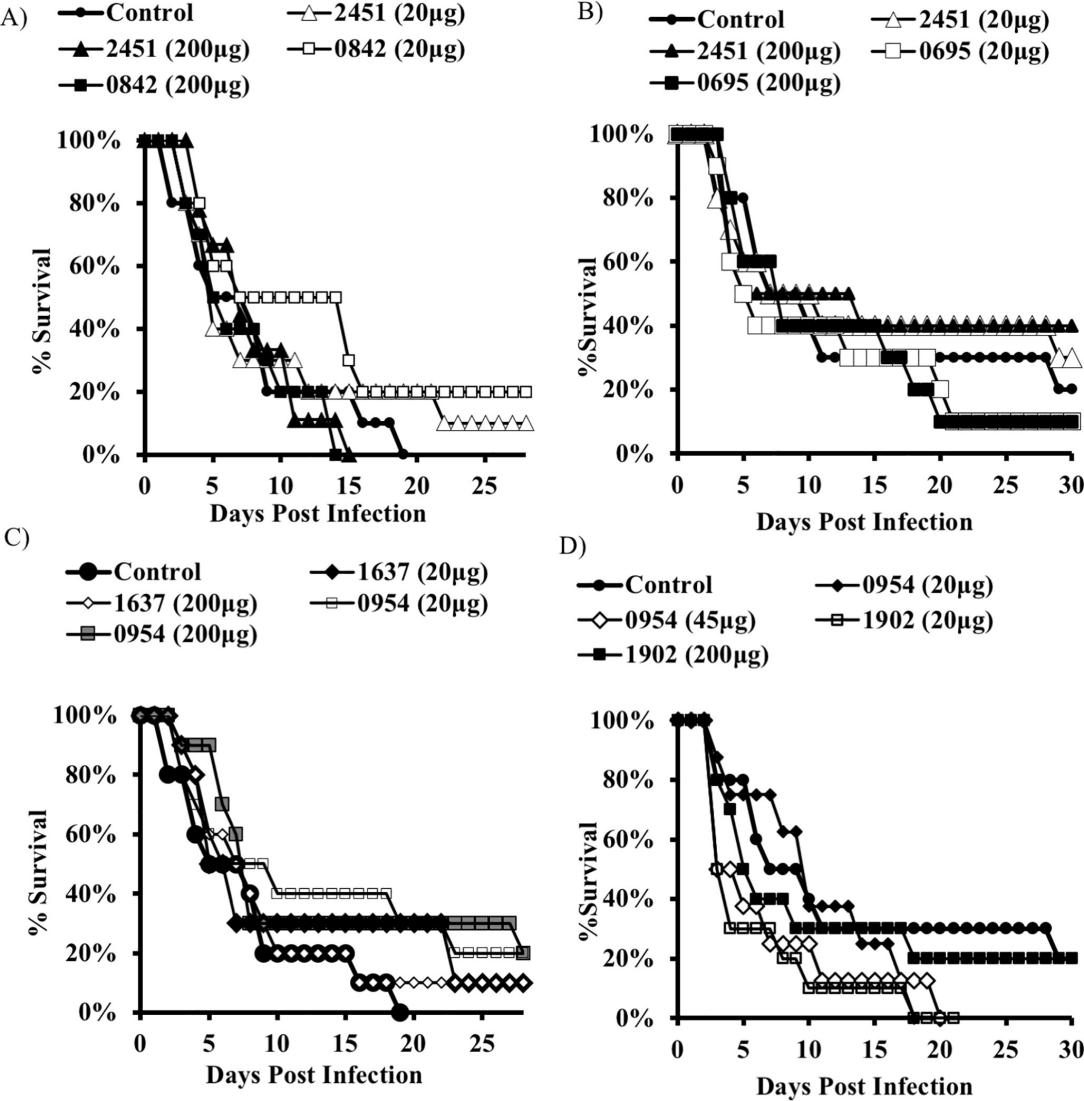
Dose optimization. **A, B)** Bacteremia model. BALB/c mice (n = 8) were immunized with individual antigens (20 or 200 micrograms) and then challenged with a *S*. *aureus* IV infection. Mice were monitored daily and euthanized when moribund. No benefit was observed in follow up studies in the bacteremia model. Increasing the antigen dose 10-fold did not result in a statistically significant benefit.

To determine the potential for improved efficacy with dose optimization, we repeated the immunization strategy but tested both the original 20 μg and a higher 200 μg dose (except for SACOL1173, which was fully protective in the skin model at the 20 μg dose). Protection was again seen in the skin model (Fig 3), but once again not in the bacteremia model irrespective of dose (Fig 4).

### Combination vaccine

**Fig 5:**
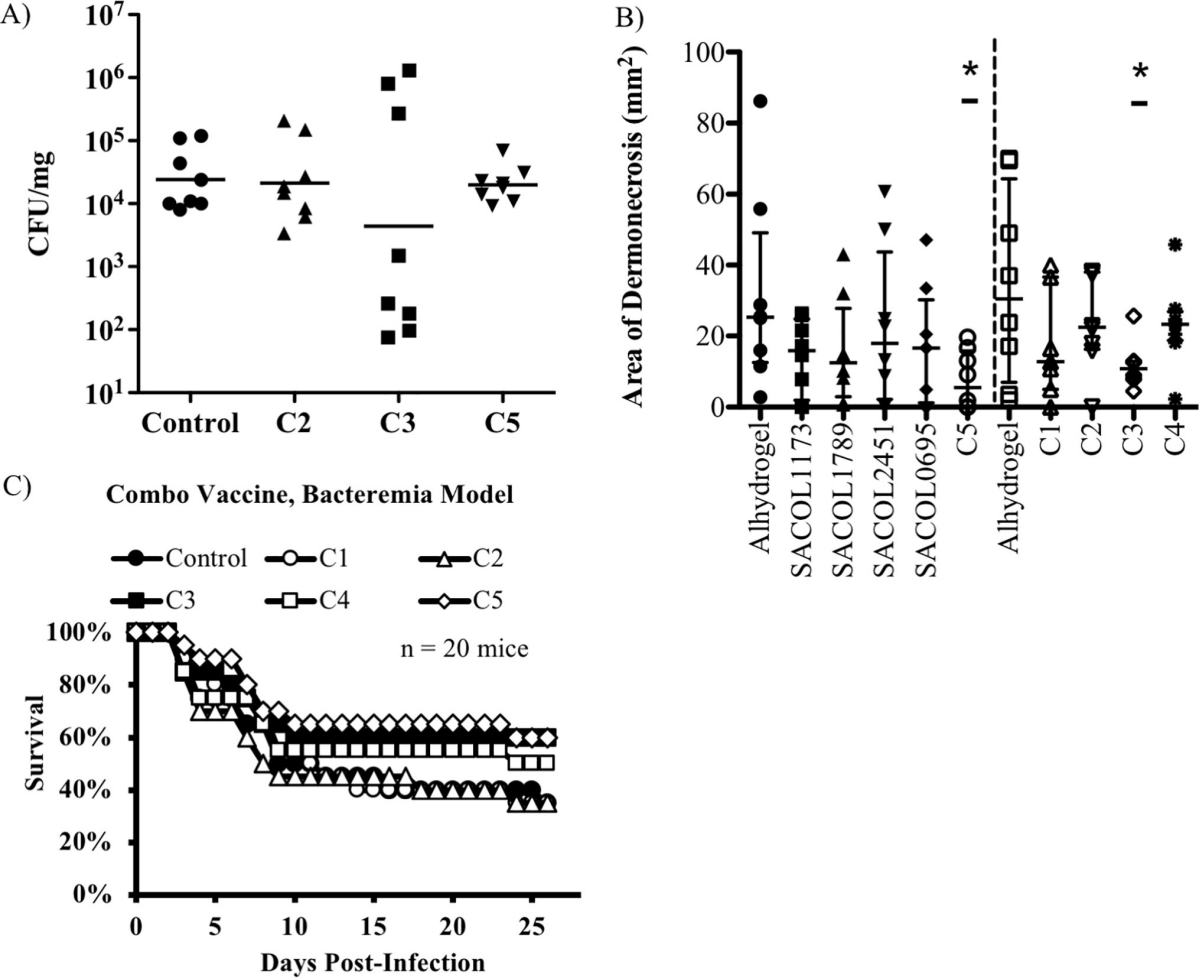
Combination vaccines. Combinations of antigens that offered protection in the bacteremia model (SACOL2451 and SACOL0695) were combined with antigens that were found to be protective in the skin model (SACOL1173 and SACOL1789). **A, B)** Skin infection model. BALB/c mice (n = 8) were immunized and then challenged with a *S*. *aureus* in a skin infection model. Combination groups “C3” and “C5” were able to reduce the area of dermonecrosis relative to the Alhydrogel control (Wilcoxon rank-sum, p < 0.05) but the reduction in CFU burden was not statistically significant (n = 8 mice per group). **C)** Bacteremia model. BALB/c mice (n = 20) were immunized with antigen combinations and then challenged with a *S*. *aureus* IV infection. Mice were monitored daily and euthanized when moribund. Combination groups "C3", "C4", and "C5" resulted in a greater number of mice surviving but this was not statistically significant (n = 20 mice per group). “C1” = SACOL1173, SACOL1789, SACOL2451, and SACOL0695; “C2” = SACOL0695, SACOL1789, and SACOL2451; “C3” = SACOL0695, SACOL1173, and SACOL2451; “C4” = SACOL1173, SACOL1789, and SACOL0695. “C5” = SACOL1173, SACOL1789, and SACOL2451. * P < 0.05, relative to the alhydrogel control (Wilcoxon rank-sum).

Thus a single antigen vaccine strategy was not feasible for protecting against both skin infection and bacteremia. Therefore, we systematically developed vaccine combinations by combining individual antigens (Table 1) that provided the best protection in each respective model. As before, we evaluated the ability of the vaccine to delay the time to moribund condition in the bacteremia model and to reduce CFU and/or lesion size in the skin model. Reduced lesion size in the skin model was observed for combinations “C3” and “C5”. However, only combination “C3” resulted in a reduction in CFUs as well. Immunization with combinations “C1” and “C2” resulted in the best numerical survival in the bacteremia model, however no group achieved statistically significant improvements in survival (Fig 5).

**Table 1:** Summary of combination immunizations.

**Combo Group**	**Description**
**C1**	SACOL1173, SACOL1789, SACOL2451, and SACOL0695
**C2**	SACOL0695, SACOL1789, and SACOL2451
**C3**	SACOL0695, SACOL1173, and SACOL2451
**C4**	SACOL1173, SACOL1789, and SACOL0695
**C5**	SACOL1173, SACOL1789, and SACOL2451

**Fig 6:**
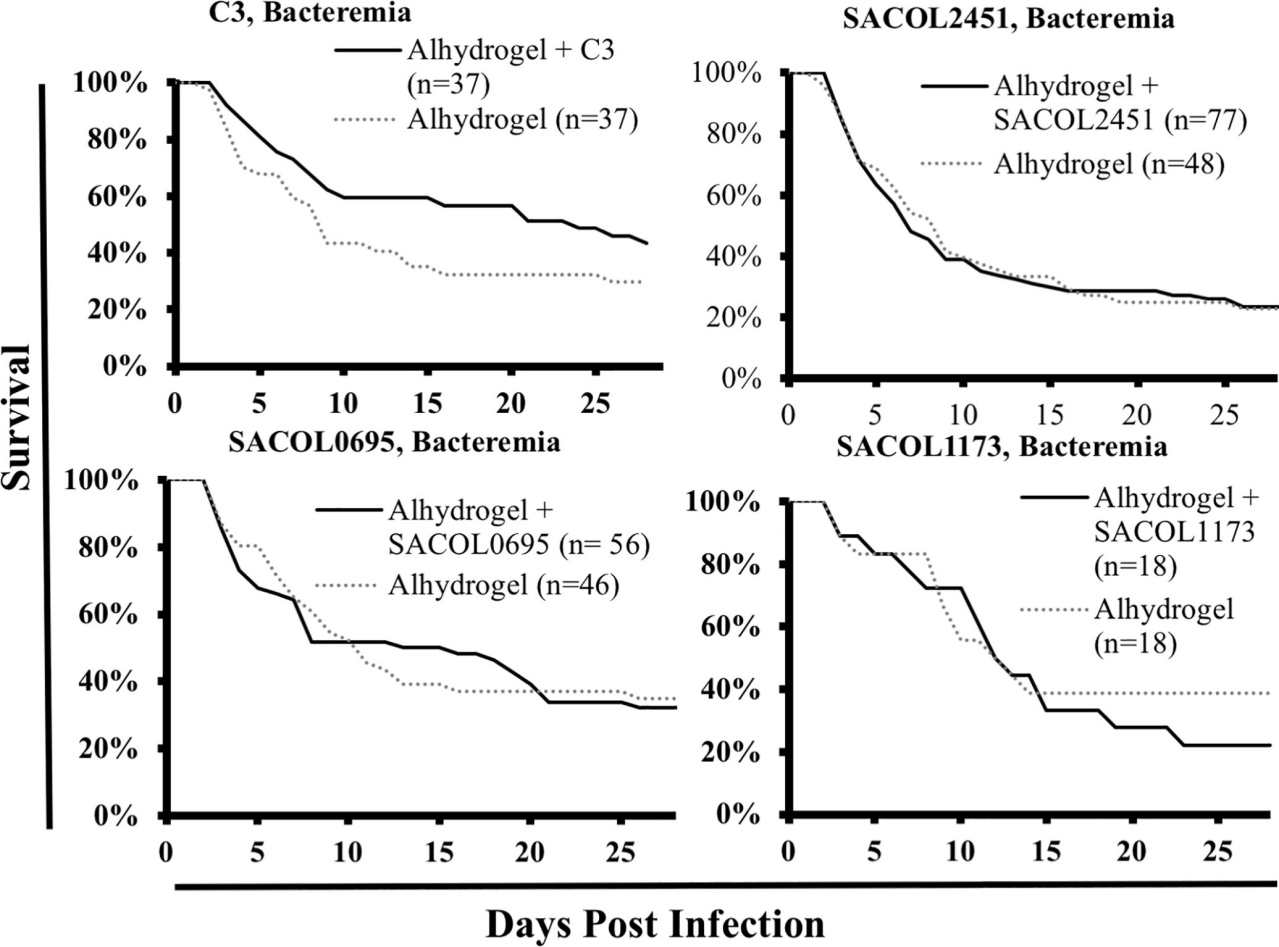
Group "C3" efficacy, bacteremia model. BALB/c mice were immunized and then challenged with a *S*. *aureus* IV infection. Mice were monitored daily and euthanized when moribund. We had previously observed mixed results when immunizing mice with the different regimens and therefore we carried out 4 independent experiments (combined n = 37 mice). Immunization and infections were standardized across experiments. No significant difference was observed between immunized and control mice. "C3" is composed of an equal mixture of SACOL1173, SACOL2451, and SACOL0695 antigens.

As group “C3” was the most promising in the skin model, we decided to repeat the bacteremia model using the components of group "C3" alone and in combination. Once again, we found no significant benefit of vaccination in the bacteremia model (Fig 6).

### Alternative adjuvants

Vaccines require the use of an adjuvant to help direct the proper host immune system to yield a protective response. All previous experiments had been carried out using Alhydrogel (aluminum hydroxide) as an adjuvant and we wanted to expand the adjuvants tested to include MPLA (which activates TLR4), Imiquimod (which activates TLR7), and/or whole glucan particles (WGP, which activates Dectin-1). Alhydrogel was also included to enable depot formations. We tested variable combinations of adjuvants with the fixed antigen combination of group "C3". We elected to test only group "C3" because it had resulted in protection in the skin model and a trend to protection in the bacteremia model. Of note, “C3” did not include SACOL1164, which performed well in individual skin infection experiments. We chose “C3” to focus more on the bacteremia model, for which protection was much more challenging to identify, particularly since SACOL1173 was completely protective in the skin model, and was included in “C3”.

**Fig 7:**
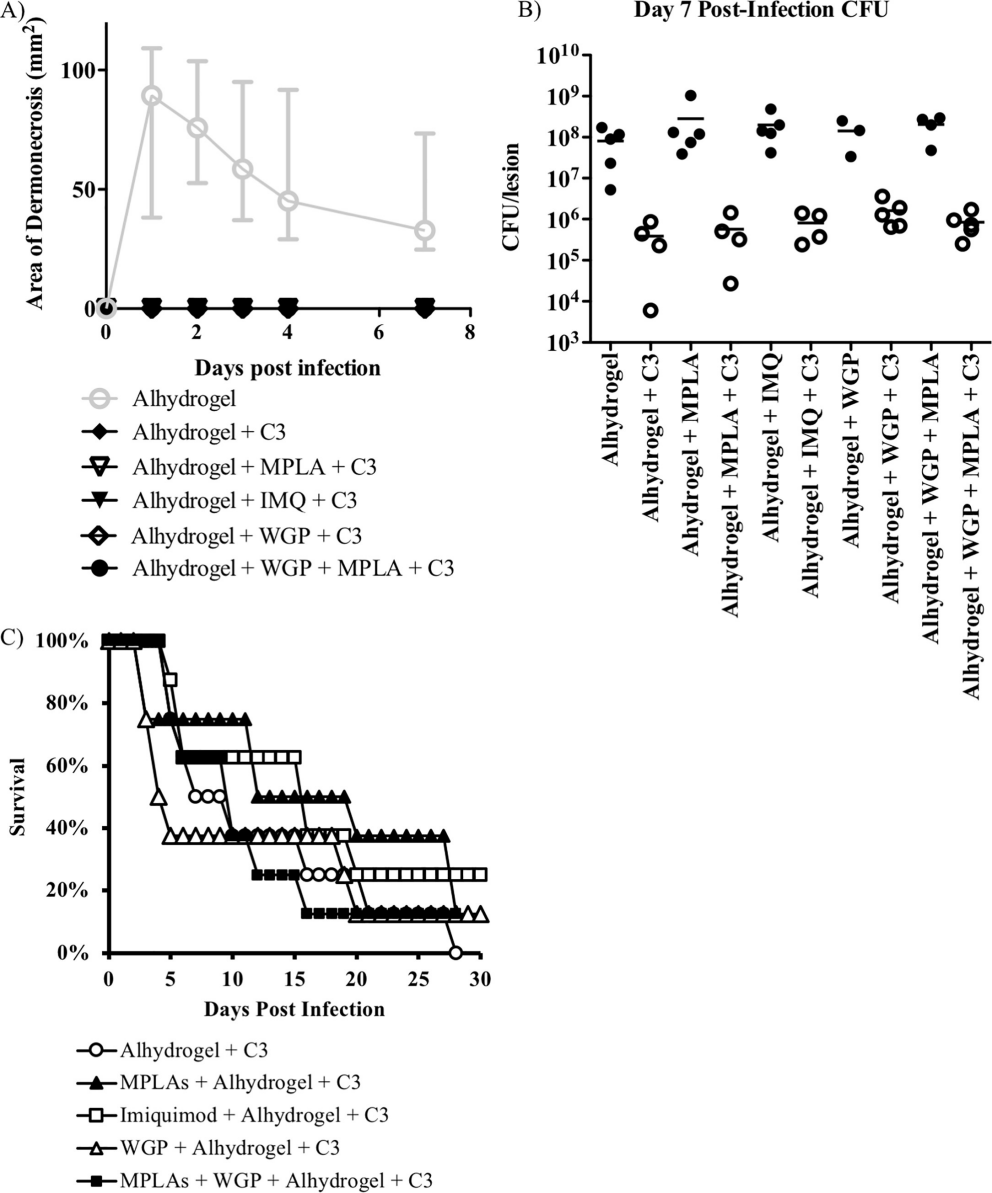
Adjuvant optimization. In attempt to improve the activity of "C3", we tried to substitute the adjuvant used. Skin infection model. BALB/c mice (n = 8) were immunized with “C3” and then challenged with a *S*. *aureus* in a skin infection model. Mice were monitored daily and the wound size was recorded. For the bacteremia model, BALB/c mice were immunized and then challenged with a *S*. *aureus* IV infection. Mice were monitored daily and euthanized when moribund. **(A)** A significant benefit (P <0.05, nonparametric log-rank test) was observed for both the prevention of dermonecrosis. **(B)** A significant reduction in CFU burden relative to the adjuvant only control (P < 0.05, Wilcoxon rank-sum) was also observed. All vaccine formulations that contained "C3" performed similarly. This is in contrast to the limited observed benefit in the bacteremia model **(C)**. "C3" = SACOL0695, SACOL1173, and SACOL2451. n = 8 mice per group.

We again observed that mice immunized with the combination group "C3" antigens resulted in protection in the skin model (Fig 7). Vaccine efficacy was observed for each of the various adjuvant combinations tested. However, no statistically significant difference was observed in the bacteremia model when group "C3" antigens were added to any of the adjuvant formulations (Fig 7).

## Discussion

There is an urgent need for preventative vaccines against *S*. *aureus* infections of all kinds, given their frequency, virulence, and antibiotic resistance. We have previously hypothesized that a mixture of multiple antigens would be necessary to protect against both skin and bloodstream infections [15–17]. We set about performing a systematic test of candidate staphylococcal proteins to test for vaccine efficacy, in both skin and lethal bloodstream models of infection.

We identified a number of staphylococcal protein antigens that display protective efficacy in either a bacteremia or skin model of infection. Interestingly, individual antigens that demonstrated efficacy in one disease model had little overlap with the other disease model. This led us to pursue a combination vaccination strategy. The combination was considerably more effective in the skin model, but we were not able to achieve substantial protection in the bloodstream infection model irrespective of antigen combinations or adjuvants tested.

One possible explanation for the lack of protection in both models is the use of different strains of *S*. *aureus* (both of which are USA300) for each model. However, for a vaccine to be useful, it must protect against most clinical isolates encountered, and as such, failure to protect against different isolates still makes translational development problematic. Furthermore, the proteins used are identical across the strains tested, so antigenic variation across the strains is not the explanation for lack of efficacy across both models.

Another possible explanation is that the cellular physiology of the bacteria differ at different disease sites. Other studies have also found that vaccine effectiveness will vary between disease models [19]. For example, the composition of bacterial surface antigens can differ in different host anatomical locations. It is also not necessarily true that the most abundant antigen is the most immunogenic and we therefore chose not to preselect our candidate antigens based solely on the relative abundance of the antigen present in each disease model. We took an unbiased approach and tested individual antigens for efficacy in each disease model.

The majority of work presented here utilized Alhydrogel as the adjuvant. Aluminum is an FDA approved adjuvant and is currently included the hepatitis A, hepatitis B, DTaP, Hib, and HPV vaccine formulations [20–22]. Aluminum adjuvants establish a depot formation for the sustained release of antigen after immunization, and also stimulates antigen presenting cells by activating the NALP3 inflammasome. MPL is a detoxified version of LPS and functions by activating immune cells through TLR4. Glucan can signal through multiple receptors, however the WGP product that we used functions by signaling only through Dectin-1 to stimulate macrophages and dendritic cells. Imiquimod is a TLR7 agonist and polarizes a Th1 response. The 4 adjuvants that we tested in total are not an exhaustive list and the possibility exists that the protection provided by the group "C3" antigens in the bacteremia model could be further improved if paired with a different adjuvant [21]^.^ However, there is not an obvious way to select the proper adjuvant because the optimal immune response to protect against *S*. *aureus* infections is still unknown [17,19].

In summary, this combinatorial approach to vaccine testing underscores the ongoing challenges of vaccine development targeting *S*. *aureus*. Antigens that are protective at one anatomical site of infection may well not be effective at protecting against infection at other sites. We have no established surrogate marker to predict in vivo efficacy. Furthermore, the immunological mechanisms of protection may differ at different sites of infection. Further research is needed to identify antigens that are broadly protective *in vivo*, and adjuvants that will enhance protection at various sites of infection.

## Methods

All animal experiments were approved by the Institutional Committee on the Use and Care of Animals at the USC Keck School of Medicine and the University of Chicago, following the National Institutes of Health guidelines for animal housing and care. Mice were housed in standard cages at a maximum density of five per cage, and given food and water ad libitum. Mice were euthanized by CO_2_ followed by cervical dislocation when they reached moribund condition, defined as inability to ambulate on tactile stimulation.

### Immunization protocol

Female BALB/c mice (9–15 weeks) were purchased from Taconic. Immunizations done using a prime-boost strategy. After the initial “prime” immunization, the “boost” immunization was administered 21 days later. Mice were then challenged *S*. *aureus* 14 days after the boost immunization.

Concentrations of purified antigens were determined by BCA assay, and endotoxin levels were assessed using the Pierce LAL Chromogenic Endotoxin Quantitation Kit (ThermoFisher 88282). Antigens were mixed with 0.1% Alhydrogel (Brenntag, Westbury, NY) in PBS, and injected subcutaneously in a volume of 200 μL. Mice were primed, then boosted with the same formulation.

We additionally tested adjuvant components including Imiquimod (Invivogen vac-imq), WGP Dispersible (Invivogen tlrl-wgp), and MPLA Synthetic VacciGrade (Invivogen vac-mpls). MPLAs, Imiquimod, and WGP were administered at 10, 50, and 100 μg per dose respectively.

### Antigens

To identify antigens against which immune responses are generated during infection, we inoculated mice with a sublethal dose of *S*. *aureus* LAC (USA300). We bled the mice prior to immunization and then again at 14 days post-immunization to harvest immune serum. We used lysostaphin cell surface preparations[23,24] of the *S*. *aureus* to run 2D gel Western blots (size vs. isoelectric focusing) as we have previously described.[18] The immune serum identified several novel antigens that were not recognized by pre-immune sera. Cell wall protein antigens were identified by Matrix-Assisted Laser Desorption/Ionization Time-of-Flight Mass-Spectrometry (MALDI-TOF) analysis. None had previously been assessed in vaccine studies. These antigens were: SACOL1637, SACOL0695, and SACOL0954 (Table 2). The cDNAs encoding these antigens were cloned into the expression vector pQE-1 (Qiagen) and expressed by Isopropyl β-D-1-thiogalactopyranoside (IPTG) induction, followed by His-tag protein purification. Endotoxin was removed from protein preparations using a Detoxigel endotoxin removal kit (Pierce) or ToxinEraser kit (GenScript). Final endotoxin levels were less than 5 EU per immunization dose.

**Table 2:** Summary of the antigens tested.

**Gene**	**Symbol**	**Description**	**Source**
**SACOL0139**	Cap5E	Capsular polysaccharides 5E subunit	Jean Lee
**SACOL0452**	-	Alkyl hydroperoxide reductase	Merck
**SACOL0688**	-	Mg/Zn transport system protein	Merck
**SACOL0695**	ABC2	ABC transporter protein 2	This study
**SACOL0842**	Eno	Enolase	Merck
**SACOL0856**	ClfA	Clumping factor A	Merck
**SACOL0912**	-	Hypothetical protein	Merck
**SACOL0954**	EfTu	Elongation Factor	This study
**SACOL1016**	FABI	Enoyl-(acyl-carrier-protein) reductase	This study
**SACOL1164**	Ecb	Extracellular complement binding protein	Merck
**SACOL1173**	Hla	α-hemolysin	Merck
**SACOL1637**	Hsp70	Heat shock protein 70	Merck
**SACOL1789**	Mce	Mammalian cell entry protein	Merck
**SACOL1902**	-	Hypothetical protein	Merck
**SACOL2074**	-	D-alanyl-alanine synthetase	Merck
**SACOL2451**	-	ABC Transporter	Merck
**SACOL2493**	-	Hypothetical protein	Merck
**SACOL2493**	-	Hypothetical protein	Merck

In addition, Merck provided a panel of recombinant protein antigens (Table 2) that had previously displayed protective efficacy in their internal testing, and that also were immunogenic in macaques as determined by a Th17 Enzyme-Linked ImmunoSpot (ELISPOT) assay.

### Bacteremia infection model

We re-struck fresh cultures of the USA300 strain *S*. *aureus* LAC (Los Angeles County Jail) from frozen stock monthly to ensure no loss of virulence. For the bacteremia infection model. Overnight cultures of LAC were subcultured 1:100 in fresh tryptic soy broth on the day of challenge, and harvested 3 hours later at OD_600_ of 0.5. Bacterial cells were pelleted by centrifugation and washed three times, resuspended in sterile PBS at a density of 7E7 CFU/200 μL, and injected intravenously into the tail vein of pre-warmed mice. Inocula were confirmed by plating serial dilutions on tryptic soy agar and counting colonies. Mice were followed for >28 days for signs of morbidity; mice that were moribund were euthanized according to IACUC guidelines.

### Skin infection model

Skin infections were done as previously described.[25] For skin infections we used the USA300 clinical isolate SF8300 (provided by Henry Chambers, University of California, San Francisco).[26] Overnight cultures of SF8300 were subcultured in fresh tryptic soy broth at 1:100 on the day of challenge, and harvested 2–3 hours later at an approximate OD_600_ of 1.8. Bacterial cells were pelleted by centrifugation, washed, and resuspended in sterile PBS at a density of 1.5 x 10^7^ CFU/50μL. Inocula were confirmed by plating serial dilutions on tryptic soy agar and counting colonies.

Mice were sedated, and their flanks were shaved and disinfected. Mice were injected subcutaneously with 50 μl of the *S*. *aureus* suspension at a concentration of 1.5 × 10^7^ CFU/50 μl. Skin lesions were photographed each day for 15 days using a 100-mm^2^ square as a standard. The size of the lesion was measured using Adobe Photoshop software. To determine the bacterial burden, skin lesions were excised on day 7 post-infection. Serial dilutions of the tissue homogenates were plated on mannitol salt agar to quantify the bacterial load. For the lethal sepsis model, mice were challenged by tail vein injection of a 250-μl suspension of *S*. *aureus* of 2.5 × 10^8^ CFU/mL and monitored for survival up to 28 days. At 24 hours post-infection, blood collected from tail vein is diluted and plated on mannitol salt agar to quantify bacterial burden.

Subcutaneous inoculation of mice on the flank with a USA300 CA-MRSA strain (SF8300) results in the formation of localized dermonecrotic lesions in 100% of mice. These lesions initially increase in size, before eventual spontaneous clearance by naïve animals. Control mice developed lesions that reached a maximal size within 1 day of challenge and then steadily decreased in size over the following two weeks. Mice that showed signs of immunity either developed lesions that were initially much smaller than those on control mice, had lesions that decreased in size more rapidly, or both.

### Statistical analysis

Survival was compared by the nonparametric log-rank test. Surface staining and bacterial killing were compared with the Wilcoxon rank-sum test for unpaired comparisons. Group comparisons were done using the Wilcoxon rank-sum test. Differences were considered significant if the P value was < .05. All statistics were run using KyPlot software.

## Supporting information

S1 Raw Data Raw data. (ZIP)
